# Chitosan/Gelatin Scaffolds Loaded with *Jatropha mollissima* Extract as Potential Skin Tissue Engineering Materials

**DOI:** 10.3390/polym15030603

**Published:** 2023-01-24

**Authors:** Matheus Ferreira de Souza, Henrique Nunes da Silva, José Filipe Bacalhau Rodrigues, Maria Dennise Medeiros Macêdo, Wladymyr Jefferson Bacalhau de Sousa, Rossemberg Cardoso Barbosa, Marcus Vinícius Lia Fook

**Affiliations:** 1 Postgraduate Program in Materials Science and Engineering, Department of Materials Engineering, Federal University of Campina Grande, Campina Grande 58429-900, PB, Brazil; 2 Department of Materials Engineering, Federal University of Campina Grande, Campina Grande 58429-900, PB, Brazil

**Keywords:** chitosan, gelatin, *Jatropha mollissima*, scaffolds

## Abstract

This work aimed to develop chitosan/gelatin scaffolds loaded with ethanolic extract of *Jatropha mollissima* (EEJM) to evaluate the influence of its content on the properties of these structures. The scaffolds were prepared by freeze-drying, with different EEJM contents (0–10% (*w*/*w*)) and crosslinked with genipin (0.5% (*w*/*w*)). The EEJM were characterized through High Performance Liquid Chromatography coupled to a Diode Array Detector (HPLC-DAD), and the determination of three secondary metabolites contents was accomplished. The physical, chemical and biological properties of the scaffolds were investigated. From the HPLC-DAD, six main substances were evidenced, and from the quantification of the total concentration, the condensed tannins were the highest (431.68 ± 33.43 mg·g^−1^). Spectroscopy showed good mixing between the scaffolds’ components. Adding and increasing the EEJM content did not significantly influence the properties of swelling and porosity, but did affect the biodegradation and average pore size. The enzymatic biodegradation test showed a maximum weight loss of 42.89 within 28 days and reinforced the efficiency of genipin in crosslinking chitosan-based materials. The addition of the extract promoted the average pore sizes at a range of 138.44–227.67 µm, which is compatible with those reported for skin regeneration. All of the scaffolds proved to be biocompatible for L929 cells, supporting their potential application as skin tissue engineering materials.

## 1. Introduction

Tissue engineering aims to develop and use biomaterials, such as scaffolds, to support adequate tissue regeneration and repair. These are structures with pores of varying sizes and shapes, interconnected or not. Such characteristics are defined depending on the cell type of the tissue or organ to which a scaffold will be applied [1,2,3].

Among the biomaterials investigated for the development of scaffolds for the treatment of skin lesions, natural polymers or biopolymers (hyaluronic acid, collagen, chitin, chitosan, among others) have played an adequate role due to their biocompatibility, biodegradability, and similarity with the macromolecules recognized by the human body, making them excellent mimics of extracellular matrix (ECM) systems [4]. The combination of polysaccharide and protein, such as chitosan and gelatin, respectively, proved to be a functional strategy to mimic the native ECM, and are, therefore, an excellent alternative for the development of scaffolds for tissue engineering [5,6,7].

Chitosan is an abundant polysaccharide with properties that include antibacterial action, biodegradability, and cytocompatibility [8,9,10]. However, the low mechanical strength of pure chitosan-based biomaterials limits their application. In this sense, to optimize their mechanical properties, the use of crosslinking agents, such as genipin, is a viable alternative [11,12,13]. Moreover, according to Si et al. [14], the effect of pure chitosan in promoting cell proliferation is limited due to the lack of clues to induce cell migration and differentiation on the surface of scaffolds. In this sense, the development of chitosan-based scaffolds in combination with other biopolymers, such as gelatin, is reported.

Blending chitosan with gelatin is effective as the protein contains an Arg–Gly–Asp (RGD)-like sequence that promotes cell adhesion and migration and forms a polyelectrolyte complex with such polysaccharide [15,16,17,18]. Xu et al. [19] developed scaffolds based on chitosan, gelatin and decellularized extracellular matrix by freeze-drying with high biocompatibility, as well as good antibacterial activity and the appropriate mechanical properties for skin tissue engineering. Zhang et al. [20] built sandwich-like scaffolds of chitosan, gelatin and polycaprolactone by electrospinning and lyophilization; the resulting product had good biocompatibility and was capable of accelerating blood clotting for guided periodontal tissue regeneration. Casimiro et al. [21] conducted in vitro and in vivo studies for scaffolds based on chitosan, gelatin and polyvinyl alcohol obtained by freeze-drying and ionizing radiation techniques, the results obtained, with good vascularization and a faster healing process, set these materials as a promising potential as a new alternative for skin wound healing. Furthermore, in order to develop new biomaterials and investigate the potentiality and properties of new substances for tissue engineering, studies on the incorporation of natural derivatives in chitosan/gelatin structures, such as essential oils and plant extracts, have been carried out [22,23,24].

The latex and extracts of *Jatropha molissima* (Pohl) Bail, a plant of the Euphorbiaceae family popularly known in Brazil as “*pinhão-bravo*”, are widely used in folk medicine, mainly for promoting anti-inflammatory, antibacterial and antioxidant action resulting from secondary metabolites [25]. Dias et al. [26] evaluated the antioxidant and cytogenotoxic potential of hexane (HE), ethyl acetate (EA) and methanol (ME) extracts from “*pinhão-bravo*” leaves and concluded that the ME extract is promising as an antioxidant as it showed no cytogenotoxic effect. Although the HE and EA extracts did not show cytogenotoxic activity, they inhibited cell division in Allium cepa cells at all of the evaluated concentrations, which may be promising for antitumor activity studies. Queiroz Neto et al. [27] investigated the chemical composition and pharmacological activities of *J. molissima* derivatives. It was observed that they have good antioxidant activity and that, even at low concentrations, both its latex and extracts inhibit the growth of gram (+) and gram (-) bacteria, evidencing the antibacterial property of the compounds, attributed to the presence of secondary metabolites.

Although the properties of *J. molissima* derivatives have already been studied, their application in the development of tissue engineering materials has not yet been reported, as well as the effects of their combination with chitosan and gelatin for the potential treatment of wounds. In this sense, this work aims to develop scaffolds based on chitosan, gelatin and ethanolic extract of *Jatropha mollissima* (EEJM). The EEJM and scaffolds’ properties are studied.

## 2. Materials and Methods

### 2.1. Materials

Chitosan of high molecular weight and degree of deacetylation of ~80% was provided by the Northeastern Biomaterials Evaluation and Development Laboratory–CERTBIO (Campina Grande, PB, Brazil). Gelatin from porcine skin, phosphate-buffered saline (PBS), lysozyme and genipin were purchased from Sigma-Aldrich^®^, Merck Group (Darmstadt, Germany). Acetic acid and absolute ethyl alcohol were purchased from Neon^®^ (Suzano, –SP, Brazil). The latex of *Jatropha mollissima* (Genetic Heritage No. AD5B98B) was collected at Sítio Valente, located in the city of Boa Vista–PB, Brazil (Lat 7° 20’ 18.29” S Long 36°14’ 17.11” W).

### 2.2. Methods

#### 2.2.1. Development of the Ethanolic Extract of *Jatropha mollissima*

The procedures for the identification, collection and extraction of the plant sample of J. molíssima were carried out according to the methodology of Ribeiro et al. [28]. Initially, the botanical identification was performed at the Manuel Arruda Câmara Herbarium (ACAM) of the State University of Paraíba–UEPB. After selecting the specimen, based on their harmful action on the plant stem, the latex was collected in sterile tubes wrapped in aluminium foil. Then, the material was kept under refrigeration (approximately 5 °C) and protected from light until the next step.

The ethanolic extract of *J. mollissima* was developed based on the methodology described by Dantas et al. [29]. First, the raw latex was mixed with absolute ethyl alcohol in a 1:1 ratio, conditioned at room temperature and protected from light for three days, using 1 (one) liter of latex to evaluate the process yield. Then, the mixture was subjected to vacuum filtration and rotary evaporation at 50 °C and 30 rpm. Finally, the liquid was frozen in an ultra-freezer (−80 °C) for 24 h and subjected to lyophilization for 72 h in a LIOTOP model L108 lyophilizer (São Carlos, São Paulo, Brazil). After the end of the process, the EEJM powder was stored under ambient conditions.

#### 2.2.2. Scaffolds Preparation

The scaffolds were prepared by solution mixing. Initially, solutions of 2% *w*/*v* of chitosan (1% of acetic acid) and 2% of gelatin were developed in a *Nova Ética* model M 110-VER-4K3 mechanical shaker (Vargem Grande do Sul, São Paulo, Brazil) for one hour, the last one at 40 °C. Then, the solutions were mixed in a 70/30 ratio of chitosan/gelatin for another hour. Afterwards, the EEJM were added in the appropriate proportion (0, 1, 2.5, 5, 7.5 ou 10%), maintaining mechanical agitation for one hour and, finally, 0.5 % w/w genipin (30 min of mixing) at 40 °C. These steps were carried out using constant agitation (400 rpm). The final solutions were poured into 6-well plates, with 3 mL per well. Then, the plates were submitted to the ultra-freezer (−80 °C) for 24 h and, finally, freeze-dried for 24 h to obtain the scaffolds. The samples were neutralized in an atmosphere of ammonium hydroxide (1M), followed by washing several times with distilled water, freezing and lyophilization for 12 h. The biopolymer ratio and genipin content were defined based on previous exploratory tests. Table 1 presents the studied EEJM contents, as well as the sample coding.

### 2.3. Characterization

#### 2.3.1. High Performance Liquid Chromatography coupled to Diode Array Detector

The High Performance Liquid Chromatography coupled to the Diode Array Detector (HPLC-DAD) technique was applied to obtain the fingerprint and identify the main groups of secondary metabolites present in the EEJM. Chromatographic analyzes were performed on a Flexar series liquid chromatography system (Perkin Elmer, Waltham, USA) equipped with an automatic injector, oven, binary pump and diode array detector (DAD) (Flexar PDA Plus Detector). Ultrapure water, acetonitrile and methanol were used as solvents.

A 1% (*v*/*v*) acetic acid aqueous solution (A) and acetonitrile (B) were used as eluents. Analyzes were conducted on a C18 column (250 × 4.6 mm, 5 µm particle size) employing the gradient mode under the following conditions: 5% B, 0–1 min; 5–10% B, 1–5 min; 10–20% B, 5–14 min; 20–21% B, 14–24 min; 21–95% B, 24–29 min. In the equilibration and washing steps, 5% B for 5 min and 95% B for 10 min were used, respectively. The eluent flow was maintained at 0.480 mL.min^−1^, and 20 µL of the sample was injected. The oven temperature was 30 ± 1 °C. The wavelength of 280 nm was monitored. The total analysis time was 29 min.

The EEJM samples were prepared at a concentration of 2.5 mg.mL^−1^ in a 95/5 (*v*/*v*) water/acetonitrile solution, with the same initial mobile phase ratio. This proportion was chosen because samples prepared in the same initial proportion of mobile phase present a better separation of substances [30]. The samples and solvents were filtered through 0.45 µm membranes. Before each analysis, the HPLC-DAD system was equilibrated, and the baseline was monitored until stable. All of the solvents used were degassed by ultrasound.

#### 2.3.2. Determination of Phenolic, Flavonoid and Condensed Tannins Contents

The determination of total polyphenols in the ethanolic extract of *J. mollissima* was performed using the Folin-Ciocalteu method [31]. Initially, an aqueous mother solution of gallic acid (100 µg·mL^−1^) and its dilutions (1–40 µg·mL^−1^) were prepared. Then, 250 µL of each curve point were mixed with 2.5 mL of 2 M Folin-Ciocalteu reagent and 2 mL of 20% (*w*/*v*) sodium carbonate solution. After 30 min, readings were taken in a Spectrophotometer model UV-1800 (Shimadzu, Kyoto) at room temperature (25 °C) in a 300–1100 nm scan using a quartz cuvette with an optical path of 1 cm and 4 polished sides model Q-204. The preparation of the EEJM sample was carried out similarly to that of the gallic acid mother solution. The absorbance values of the 770 nm wavelength were recorded. The concentration of total polyphenols was expressed as a function of the equivalent concentration of gallic acid (mg GAE/g) in the dry extract. The quantification of the total flavonoids in the ethanolic extract of *J. mollissima* was performed according to the colorimetric method of aluminium chloride (AlCl_3_) [32]. There are three steps for this: preparation of the calibration curve, sample preparation, and data collection. For the calibration curve, quercetin methanolic solutions (mother solution) at 30 µg·mL^−1^, followed by their respective dilutions (3–30 µg·mL^−1^), were prepared. Then, 1.5 mL of each solution was individually mixed with 1.5 mL of 2% (*w*/*v*) aluminium chloride (AlCl_3_) solution. After 30 min, the absorbance of the solutions was recorded at a wavelength of 445 nm by means of a Spectrophotometer model UV-1800 (Shimadzu, Kyoto) at room temperature (25 °C) in a scan of 300–1100 nm using a cuvette quartz crystal with an optical path of 1 cm with 4 polished faces model Q-204. The sample preparation was carried out similarly to the quercetin stock solution. The concentration of total flavonoids was expressed as a function of the equivalent concentration of quercetin (mg QE/g) in the dry extract. The Vanillin-Hydrochloric Acid (HCl) method, reported by Palacios et al. [33], was used to determine the total concentration of condensed tannins. Initially, a methanolic stock solution of catechin (550 µg·mL^−1^) and its respective dilutions (10–500 µg·mL^−1^) were prepared. Then, 3 mL of a 4% (*w*/*v*) vanillin solution, 500 µL of the calibration curve point solutions and 1.5 mL of a 0.75 M HCl solution were transferred to test tubes and immersed in water at 22 °C for 20 min. The readings were performed in a Spectrophotometer model UV-1800 (Shimadzu, Kyoto) at room temperature (25 °C) in a scan of 300–1100 nm and using a quartz cuvette with an optical path of 1 cm with the 4 polished faces model Q -204. Sample preparation was carried out similarly to that of the catechin stock solution. The absorbance values of the 500 nm wavelength were recorded. The concentration of total condensed tannins was expressed as a function of the equivalent concentration of catechin (mg CE/g) in the dry extract. All of the measurements were performed in triplicate (*n* = 3).

#### 2.3.3. Fourier Transform Infrared Spectroscopy

The precursor materials and scaffolds were submitted to Fourier Transform Infrared Spectroscopy (FTIR) using Perkin Elmer’s Spectrum 400 equipment (Waltham, MA, USA). The FTIR technique was performed to identify the characteristic bands and the chemical groups present in the samples. The wavelength range was between 4000 and 650 cm^−1^ at the scan speed of 32 scan/min with 16 cm^−1^ resolution.

#### 2.3.4. Swelling Degree

The swelling degree (SD) of the scaffolds was evaluated in a PBS solution (pH = 7.4) in triplicate (*n* = 3). For the analysis, the samples (approximately 1 cm^2^) previously dried (60 °C) for 24 h were weighed, and then kept in a PBS solution at 37 °C for 24 h. When the determined time was reached, the samples were removed from the solution, slightly dried on a paper towel, and weighed again. The swelling degree was determined using Equation (1):

(1) $$SD\;\left(\%\right)=\frac{W-W_{0}}{W_{0}}\times 100,$$

where *W*_0_ is the initial sample weight, and *W* is the final sample weight.

#### 2.3.5. Enzymatic Biodegradation

In vitro enzymatic biodegradation analysis was evaluated by weight loss. In this sense, the scaffold samples (1 cm^2^) previously dried in an oven (60 °C) for 24 h were submerged in 10 mL of PBS solution containing 1.5 µg/mL of lysozyme and maintained at 37 ± 0.5 °C. The PBS/Lysozyme solution was changed weekly to ensure enzyme activity. After the predetermined period (7, 14, 21 and 28 days), the samples were removed from the solution, washed in distilled water, dried (60 °C) for 24 h and weighed. The weight loss was calculated with Equation (2):

(2) $$Weight\;loss\;\left(\%\right)=\frac{W_{0}-W_{t}}{W_{0}}\times 100,$$

where *W*_0_ is the initial mass of the sample and *W_t_* is the mass of the sample taken out of the solution in the specified period of time.

#### 2.3.6. Scanning Electron Microscopy

Scanning electron microscopy (SEM) was used to assess the scaffold morphology. The equipment utilized was a Hitachi model TM-1000 (Chiyoda, Tokyo, Japan) scanning electron microscope. The ImageJ software was used to analyze the porosity and the average pore size from the images obtained.

#### 2.3.7. Cytotoxicity Assay

The cytotoxic activity of the scaffolds was evaluated using the agar diffusion method according to ISO 10993-5 (2009) [34].

Initially, the L929 Mouse Fibroblast Cell Lines (ATCC NCTC clone 929, Rio de Janeiro Cell Bank, Brazil) were cultured in RPMI 1640 medium (Gibco–Invitrogen Corporation, Grand Island, USA) containing 10% fetal bovine serum (BS–Gibco, Life Technologies) and 1% antibiotic-antimycotic (Gibco, Life Technologies) in culture dishes until they reach confluence. Then, the cells were trypsinized to be subcultured in a suspension with a concentration of 1.0 × 105 viable cells per mL, with the aid of an automatic cell counter (Invitrogen–Thermo Fisher, Waltham, MA, USA). The cell suspensions were distributed in 6 well plates (4 mL/well) and incubated for 48 h. After the establishment of cell cultures, the microplate culture medium was aspirated, and 1 mL of the cover medium (1.8% agar, 0.01% neutral red dye added and 2× concentrated MEM) was added in each well. The plates remained in the laminar flow hood for 10 min, protected from light, waiting for the agar to solidify at room temperature. The samples and controls were placed in contact with the solidified surface of the agar, in the center of each plate, in triplicate cultures. Thus, the plates were incubated and protected with aluminium foil to avoid cellular damage by the photoactivation of neutral red (ISO 10993-5) for at least 24 h in an oven at 37 °C, humidified with 5 ± 1% CO_2_ for cytotoxicity assessment. The presence of discoloration zones and cell lysis was evaluated using a NIKON ECLIPSE TS100 (Minato, Tokyo, Japan) inverted digital microscope.

#### 2.3.8. Statistical Analysis

The statistical analysis of the data was performed by One-Way Analysis of Variance (ANOVA) and Tukey tests for the differences in means. The confidence level adopted will be 95%, and the significance level (α) was 0.005. For p-values greater than α, the difference between the means will be considered statistically insignificant. On the other hand, for p-values less than or equal to α, the difference between the means will be considered statistically significant.

## 3. Results and Discussion

### 3.1. High Performance Liquid Chromatography coupled to Diode Array Detector

**Figure 1:**
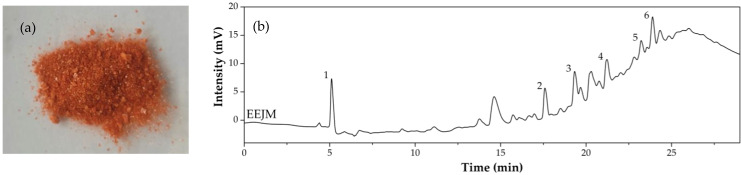
(**a**) EEJM powder appearance and (**b**) EEJM *fingerprint* chromatogram in 280 nm for identification of secondary metabolites.

The development process of the *Jatropha mollissima* ethanolic extract was successfully carried out. A yield of approximately 115 g of EEJM powder (Figure 1a) was achieved for 1 L of latex. In this sense, the HPLC-DAD technique was applied in the evaluation of the EEJM (Figure 1b).

According to Figure 1b, the separation of six significant substances can be observed. These substances were identified at a wavelength of 280 nm, which is characteristic of tannins [35], ellagitannins, phenolic compounds [36], iridoids [37], flavonoids, flavones and flavonols [38], among others. Plants of the genus *Jatropha* are known to have various classes of secondary metabolites in their composition [39]. Studies have indicated the presence of flavonoids, alkaloids, coumarins and lignins in *J. gossypiifolia* and *J. curcas* [39,40]. Other studies have shown the presence of flavonoids, polyphenols, saponins and condensed tannins in the extract of *J. curcas* leaves [41], and flavonoids and polyphenols in the ethyl acetate fraction of leaves and roots of *J. macranta* [42]. Other authors have also reported similar results [43,44,45,46], indicating that they align with the results found here.

### 3.2. Total Concentration of Phenols, Flavonoids and Condensed Tannins

The concentration of secondary metabolite classes is directly linked to the biological activity of the plant derivatives. The higher the concentration of metabolites, the greater the biological activity of the extracts. For this reason, quantifying the main classes of compounds present in plant extracts is necessary. In Table 2 are the total concentrations of polyphenols, flavonoids and condensed tannins that were quantified in the EEJM.

The results in Table 2 show that three classes of compounds, polyphenols, flavonoids and condensed tannins are present in the EEJM. The condensed tannins had the highest concentration, followed by the polyphenols and flavonoids. In the literature, research has been conducted to determine the concentration of secondary metabolites in plants of the genus *Jatropha*. Concentrations of phenols in the range of 400 to 150 μg·mL^−1^, flavonoids of 6 to 2 μg·mL^−1^ and tannins of 35 to 10 μg·mL^−1^ were identified in the aqueous, ethanolic, methanolic and ketone extracts of the seeds, roots, leaves, flowers and bark of *J. curcas* [41]. In studies by Fröhlich et al. [47], the total concentration of phenols, flavonoids and tannins in the crude extract and the dichloromethane, acetate and butanolic fractions of the roots of *J. isabellei* was determined. In their results, the acetate fraction showed the highest concentrations, with 685 μg·mg^−1^ for phenols, 25.90 μg·mg^−1^ for flavonoids and 15.63 μg·mg^−1^ for tannins. Tinco-Jayo et al. [42] estimated the concentration of phenolics in the range of 359 and 306 mg·g^-1^ and flavonoids in the range of 101–23.7 mg·g^−1^ in the ethyl acetate fraction of *J. macrantha* leaves.

Only a few studies have reported on the quantification of secondary metabolites in *J. mollissima*. In addition, there are still no studies that have determined the concentration of flavonoids and condensed tannins in the latex of *J. mollissima*. The existing studies focus on determining the concentration of phenols and flavonoids in its leaves and latex. For phenols, values of 245 μg·mg^−1^ in *J. mollissima* leaf extract [26] and 58.07 mg·g^−1^ in its latex [27] are reported; these results differ from the results from the concentrations obtained here. Such differences can be explained by the difference in the parts of the plant used to prepare the extracts (leaves, flowers, bark, roots and latex) [48,49] and due to the vegetative cycle, botanical origin and climatic factors [50]. Therefore, the results confirmed the presence of the class of phenols, flavonoids and tannins metabolites in the EEJM, highlighting the tannins which presented a concentration of 431.68 mg·g^-1^ and may be one of the main groups of compounds responsible for the biological activities of *J. mollissima* latex.

### 3.3. Fourier Transform Infrared Spectroscopy

**Figure 2:**
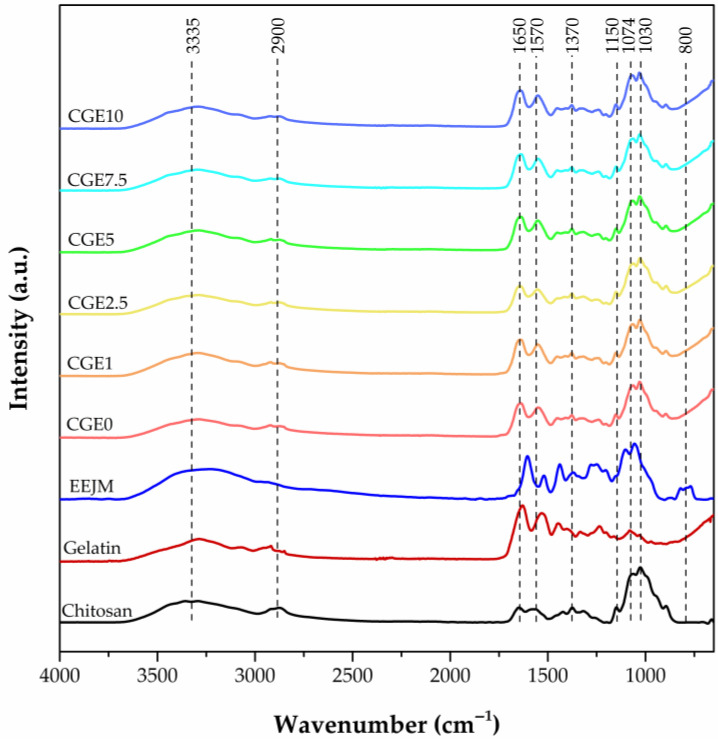
Scaffolds and precursor materials characteristic bands obtained by FTIR.

The FTIR analyses for the scaffolds and precursor materials are shown in Figure 2. The obtained spectra present characteristic absorption bands for organic compounds.

In the chitosan spectrum, the band around 3335 cm^−1^ is associated with the stretching vibration of the OH and NH bonds. The peaks at 2900 and 1316 cm^−1^ are attributed to the symmetric and asymmetric stretching vibrations of the CH groups. The peaks at 1650 and 1570 cm^−1^ are characteristic amide I and II bands. The peaks at 1370 cm^−1^ and 1258 cm^−1^ are associated with the C-OH groups’ vibration and C-O bond stretching, respectively. The bands at 1150, 1074 and 1030 cm^−1^ are attributed to the C-O-C bond of the glycosidic bonds [51,52,53,54].

In the gelatin spectrum, the band at 3290 cm^−1^ corresponds to the stretching vibration of the hydroxyl groups (OH); the band at 2932 cm^−1^ is attributed to the CH2 asymmetric stretching vibration; the band at 1628 cm^-1^ is attributed to the (C=O) stretching vibration; the band at 1530 cm^−1^ is attributed to the N-H bending vibration; and the band at 1444 cm^−1^ is attributed to the symmetrical stretching of the -COOH groups, confirming the protein molecule [51,54,55].

The EEJM Spectrum shows a broad band at 3252 cm^−1^, which is related to the stretching of the binding of the hydroxyl groups of polyphenol compounds present in the extract [56,57,58]. Superimposed on this one, the band at 2972 cm^−1^ refers to the stretching vibrations CH, CH_2_ and CH_3_ of the alkanes and alkyl groups derived from carbohydrates and sugars [59,60]. The bands located at 1604, 1522 and 1438 cm^−1^ are attributed to the elongation of the C=C-C aromatic bonds [61]. The band with a peak at 1370 cm^−1^ refers to the stretching of the C-O bond and may also be associated with the bending vibrations of the OH bonds [62]. In consonance, the bands 1106 and 1058 cm^−1^ are related to the axial deformation of the C-O bonds, which is typical of esters, alcohols, ethers and carboxylic acids. Finally, the bands 820–772 cm^−1^ are attributed to the CH bond deformation vibrations [63]. There is no absorbance between the region 2220–2260 cm^−1^, indicating that there is no presence of cyanide groups (-C≡N) in the extract [64]. All of the compounds observed from the EEJM FTIR analysis belong to secondary metabolites and corroborate the evidence found by chromatography.

The scaffold spectra show a good mixture of the components in all of the developed compositions as the characteristic bands of chitosan, gelatin and EEJM can be observed. The chitosan/gelatin interaction might be a result of the partial conversion of the electrostatic bonds into chemical by hydrogen bonding, resulting from the interaction of the protonated amine of the chitosan and the carboxyl groups of the gelatin [54].

### 3.4. Swelling Degree

**Figure 3:**
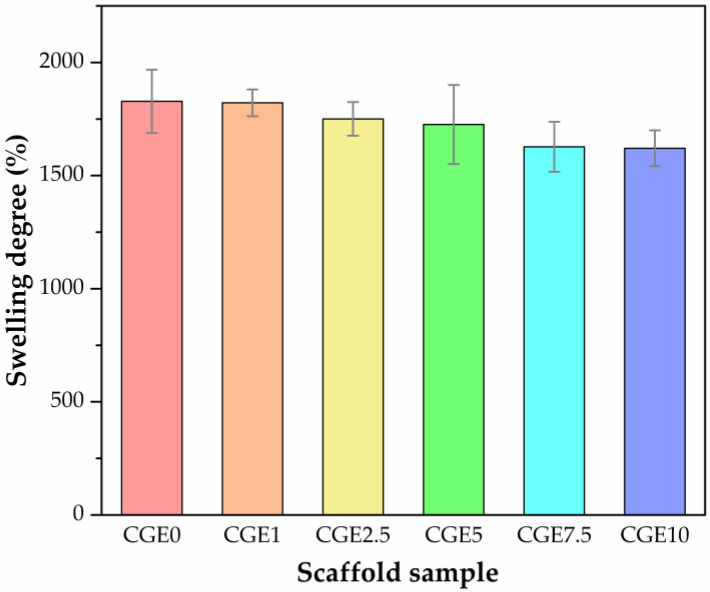
Swelling degree of scaffolds (*n* = 3).

Swelling is a fundamental property for scaffolds in tissue engineering as it influences cell migration and the diffusion of nutrients on the scaffold [65]. Skin scaffolds should absorb wound exudates and keep the wound moist, promoting a faster and more effective healing process [66,67,68]. Concerning the development of dressings for the treatment of wounds, an inadequate SD can result in the accumulation of exudate, which, although essential for healing, can cause maceration of healthy tissues, resulting in a chronic wound [69,70]. Such observations justify the importance of crosslinking chitosan-based materials in modulating properties for wound healing applications. In Figure 3, the swelling scaffolds degree can be seen.

Considerably high SD values were reached (Figure 3), and a profile of a decrease in the property could also be observed with the increase in EEJM content in the scaffold, reaching, for CGE10, an average SD of 1620.89 ± 79.01. However, according to the Tukey’s test, the means of the SD do not differ statistically (*p*-value = 0.168). Once the samples have been neutralized, the high SD value can be associated with the porous structure and gelatin content due to the hydrophilic character of such biopolymer [71]. The authors of other studies report even higher values than those found here for chitosan/gelatin scaffolds [72]. This suggests that crosslinking with genipin occurred as it induces the formation of more crosslinks in the scaffolds, in turn decreasing the SD [73]. As for the decrease in the property with the increase in the EEJM content, this may be associated with the fact that the solution becomes more concentrated or that the addition of the extract has promoted a slightly less hydrophilic character to the composition.

### 3.5. Enzymatic Biodegradation

Enzymatic degradation perfectly simulates in vivo and clinical situations [74]. Lysozyme, an enzyme present in the body, promotes the enzymatic depolymerization of chitosan through the hydrolysis of the glycosidic bonds in its subunits, N-acetyl-glucosamine and glucosamine [75,76]. These amino-monosaccharides can be absorbed as they have chemical similarities with the components of the extracellular matrix (ECM) of soft tissues [77,78,79]. The absorption of glucosamine and its derivatives stimulate the synthesis of hyaluronic acid, collagen and other ECM glycosaminoglycans, in addition to promoting hydration and anti-inflammatory activity; these are properties that contribute to the success of the scaffold by supporting cell growth and proliferation in the engineering of the new tissue [80,81,82]. Gelatin, which is a product obtained from the thermal and chemical degradation of collagen, when placed in a physiological environment, undergoes enzymatic and hydrolysis degradation [83,84,85]. Its degradation products promote the proliferation of the cells associated with wound repair and skin regeneration, such as keratinocytes, in addition to being pro-angiogenic, which favors soft tissue repair [84,86,87].

**Figure 4:**
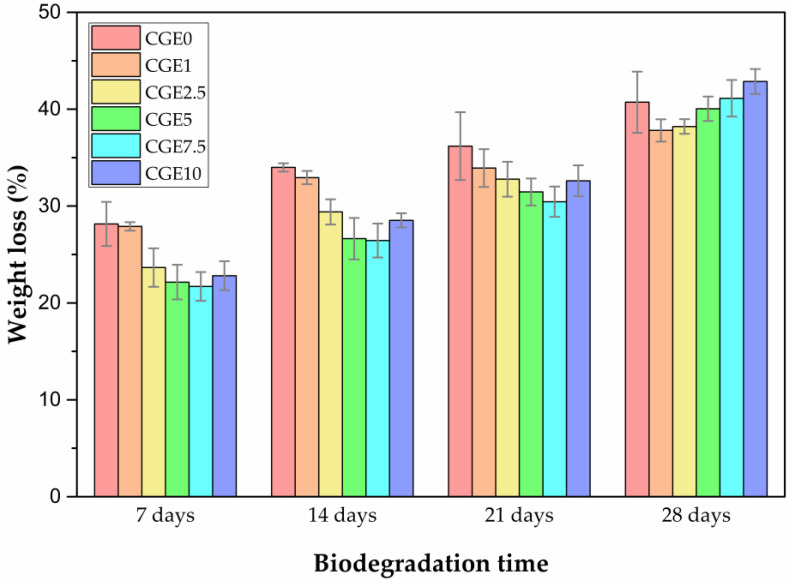
In Vitro enzymatic biodegradation of scaffolds at intervals of 7, 14, 21, and 28 days (*n* = 3).

The enzymatic biodegradation of the scaffolds (Figure 4) follows a pattern for the intervals of 7, 14, and 21 days, in which the increase in the extract content promotes a smaller weight loss, with the exception of the CGE10 sample, although this one still reaches lower values in 21 days to those obtained for CGE0 (sample without EEJM), of 32.61 ± 1.60 and 36.19 ± 6.49, respectively. After 28 days, the biodegradation profile is different, which is attributed to an increase in the breakdown of crosslinks, which makes the material (EEJM) more susceptible to degradation due to weight loss. Based on the Tukey’s test, the statistical significance of the addition of EEJM on the weight loss of the samples during the test period was confirmed (*p*-value = 0.037). It is noteworthy that in the evaluated period (28 days), the scaffolds showed a maximum mass loss of 42.89 ± 1.28% (CGE10), which can be attributed to crosslinking with genipin as the reported values of mass loss in the literature are superior to chitosan/gelatin scaffolds crosslinked with other agents [16,68].

### 3.6. Scanning Eelectron Microscopy (SEM)

**Figure 5:**
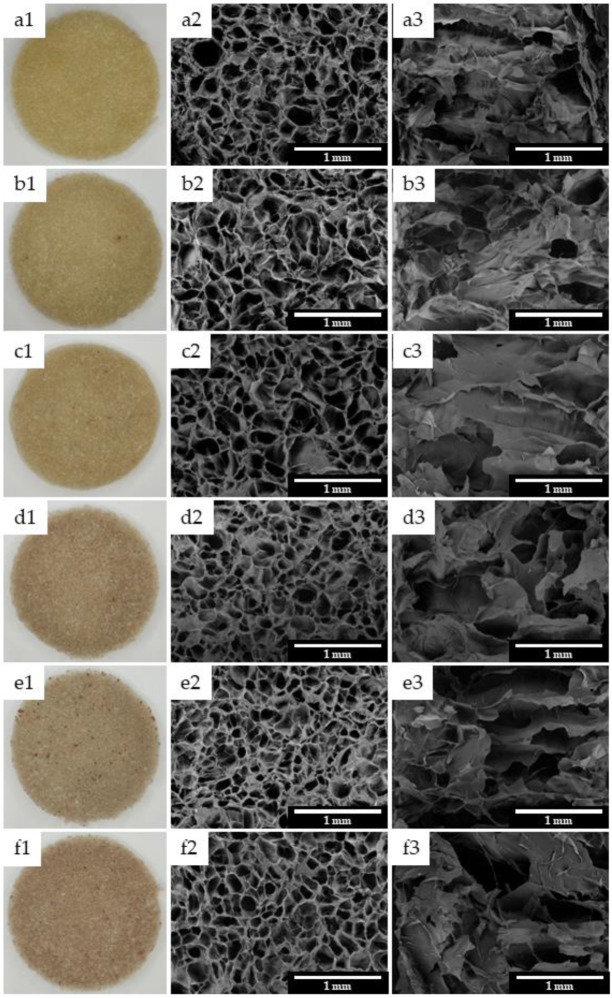
Photographs (column 1) and SEM pictures of scaffolds surface (column 2) and cross section (column 3): CGE0 (**a**), CGE1 (**b**), CGE2.5 (**c**), CGE5 (**d**), CGE7.5 (**e**) and CGE10 (**f**).

Photographs (1) and SEM images (surface (2) and cross-section (3)) of the scaffolds are shown in Figure 5. As can be seen, the EEJM content affected the color of the scaffolds, leading to its characteristic reddish color (Figure 1a). All of the samples have an open pore structure with interconnected pores characteristic of the freeze-drying method [68,88,89]. An open pore structure is essential for cell attachment and blood supply [16].

Table 3 summarizes the average porosity (%) and pore size (μm) for all of the samples.

Porosity plays a crucial role in cell migration and proliferation as it influences gas and nutrient exchange, as well as swelling [90]. The scaffolds reached average porosity values, between 57 and 60%, close to those achieved in some other studies on chitosan/gelatin scaffolds [68,72,91]. As for pore size, an average pore size in the range of 100 and 200 µm is recommended in the literature for skin tissue engineering applications [89,92]. All of the developed scaffolds reached average pore size values in the described range, with the exception of CGE0 (227.67 ± 72.06). The increase in the EEJM content in the chitosan/gelatin scaffolds tends to decrease the mean pore size and make their formats more uniform as the standard deviations decrease (Table 3). In this sense, the potential of the extract to develop structures suitable for the requirements of skin tissue engineering is highlighted.

The average porosity values showed no statistical difference (*p*-value = 0.425). On the other hand, the average pore size was significantly affected by the incorporation of EEJM (*p*-value < 0.000), according to the grouping in Table 3.

### 3.7. Cytotoxicity

**Figure 6:**
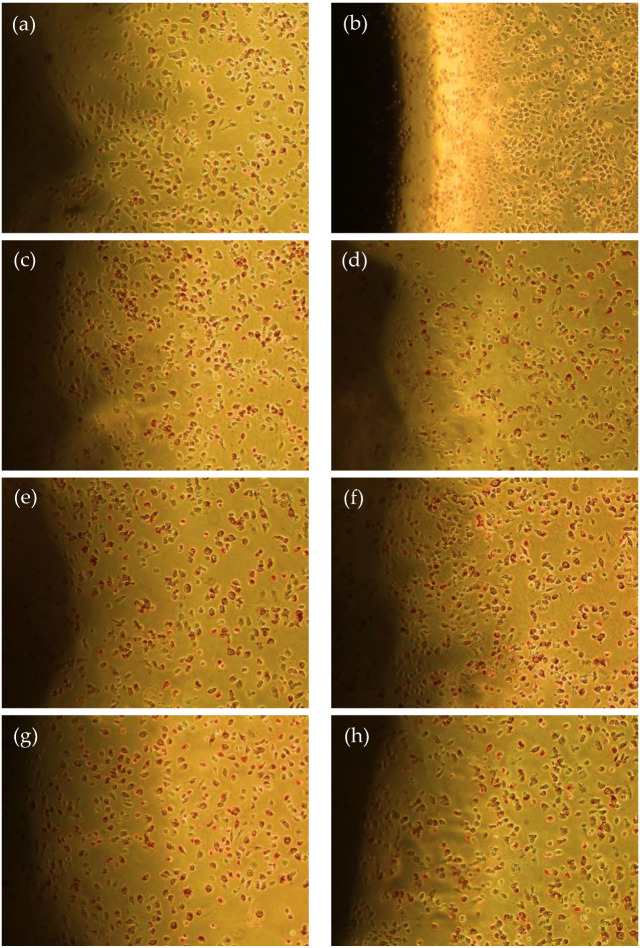
Agar diffusion test images: negative control (**a**), positive control (**b**), CGE0 (**c**), CGE1 (**d**), CGE2.5 (**e**), CGE5 (**f**), CGE7.5 (**g**) and CGE10 (**h**). All images were taken at the same magnification (100×). The shaded regions in the images are the scaffold samples.

Chitosan/gelatin scaffolds are widely reported as noncytotoxic biomaterials [19,51,55]; however, there is no evidence of the combination of these with EEJM. As previously discussed, plant extracts such as EEJM have numerous bioactive secondary metabolites, and because of their complex structure, cytotoxicity investigations are essential. In this sense, the agar diffusion test was chosen to investigate the scaffold’s cytotoxicity. The method consists of evaluating the effects of the samples through an agar layer, which protects the cells from mechanical damage and allows the diffusion of substances from polymeric samples. Table 4 presents the results obtained from the agar diffusion test, while Figure 6 presents the complementary microscopies.

The negative control, HDPE, did not promote cell lysis (Figure 6a), while for the positive control, toxic latex, cell lysis was observed as a discoloration halo was formed (Figure 6b), reaching a cytotoxicity degree of 4 (severely cytotoxic) (Table 4). There is also no formation of discoloration halos for any of the compositions of the tested scaffolds (Figure 6c–h); thus, the samples did not promote cell lysis. The results confirm that chitosan/gelatin scaffolds loaded with EEJM up to 10% (*w*/*w*) are non-toxic to L929 cells, emphasizing their potential use as skin tissue engineering materials.

## 4. Conclusions

Porous chitosan/gelatin scaffolds crosslinked with genipin and loaded with different concentrations of *Jatropha molissima* extract were successfully obtained using the freeze-drying method. *J. molissima* latex showed a yield of 115 g/L of ethanolic extract, from which six main groups of secondary metabolites were identified; among these, the phenolic, flavonoid and tannin contents were quantified by chromatography. Spectroscopy showed good mixing and interaction between the precursor materials in the scaffolds. The scaffolds showed a high swelling capacity, above 1500%, which was not affected by the extract loading, such as its porosity. On the other hand, increasing the extract content slowed down the weight loss and reduced the average pore size of the samples. Furthermore, with the addition of the extract, the average pore size was compatible with the range reported for cell adhesion and proliferation. The scaffolds with and without the addition of the extract did not show cytotoxicity, which confirms the biocompatibility of the products obtained and their potential application in skin tissue engineering.

## Figures and Tables

**Table 1:** Codes for developed scaffolds and corresponding EEJM content.

Sample Designation	Amount of EEJM (%)
CGE0	0
CGE1	1
CGE2.5	2.5
CGE5	5
CGE7.5	7.5
CGE10	10

C: chitosan; G: gelatin; E: EEJM.

**Table 2:** Total phenolic, flavonoid and condensed tannins content present in the *J. mollissima* ethanolic extract (*n* = 3).

Sample	TPC (mg GAE/g Dry Extract)	TFC (mg QE/g Dry Extract)	TTC (mg CE/g Dry Extract)
EEJM	22.91 ± 0.84	2.96 ± 0.45	431.68 ± 33.43

TPC: total phenolic content; TFC: total flavonoid content; TTC: total tannin content.

**Table 3:** Porosity and average pore size values (mean ± standard deviation) for the developed scaffolds (*n* = 3). The letters “A”, “B”, “C” and “D” are the groupings provided by Tukey’s method, in which averages that do not share letters are significantly different.

Scaffold Sample	Porosity (%)	Mean Pore Size (µm)
CGE0	59.27 ± 0.96 (A)	227.67 ± 72.06 (A)
CGE1	57.83 ± 1.73 (A)	185.86 ± 55.08 (B)
CGE2.5	58.55 ± 0.78 (A)	168.41 ± 57.74 (B, C)
CGE5	57.20 ± 1.29 (A)	165.04 ± 48.22 (B, C, D)
CGE7.5	57.41 ± 1.38 (A)	138.44 ± 34.22 (C, D)
CGE10	57.29 ± 1.89 (A)	155.20 ± 44.23 (D)

**Table 4:** Cytotoxicity classification of the samples by agar diffusion test.

Sample	Cytotoxic Degree	Definition
Negative control	0	Noncytotoxic
Positive control	4	Severely cytotoxic
CGE0	0	Noncytotoxic
CGE1	0	Noncytotoxic
CGE2.5	0	Noncytotoxic
CGE5	0	Noncytotoxic
CGE7.5	0	Noncytotoxic
CGE10	0	Noncytotoxic

## Data Availability

Not applicable.
